# High dose ionizing radiation regulates micro RNA and gene expression changes in human peripheral blood mononuclear cells

**DOI:** 10.1186/1471-2164-15-814

**Published:** 2014-09-25

**Authors:** Lucian Beer, Rudolf Seemann, Robin Ristl, Adolf Ellinger, Mohammad Mahdi Kasiri, Andreas Mitterbauer, Matthias Zimmermann, Christian Gabriel, Mariann Gyöngyösi, Walter Klepetko, Michael Mildner, Hendrik Jan Ankersmit

**Affiliations:** Department of Thoracic Surgery, Medical University of Vienna, Währinger Gürtel 18-20, 1090 Vienna, Austria; Christian Doppler Laboratory for Cardiac and Thoracic Diagnosis and Regeneration, Medical University of Vienna, Währinger Gürtel 18-20, 1090 Vienna, Austria; University Hospital of Craniomaxillofacial and Oral Surgery, Medical University of Vienna, Vienna, Austria; Center for Medical Statistics, Informatics, and Intelligent Systems, Section for Medical Statistics, Medical University of Vienna, Vienna, Austria; Department of Cell Biology and Ultrastructure Research, Center for Anatomy and Cell Biology, Medical University of Vienna, Vienna, Austria; Red Cross Blood Transfusion Service of Upper Austria, Linz, Austria; Austrian Cluster for Tissue Regeneration, Linz, Austria; Department of Cardiology, Medical University Vienna, Vienna, Austria; Department of Dermatology, Research Division of Biology and Pathobiology of the Skin, Medical University Vienna, Lazarettgasse 14, 1090 Vienna, Austria

**Keywords:** Microarray, MicroRNAs, Messenger RNA, Apoptosis, Mononuclear leukocytes, p53, Ionizing radiation

## Abstract

**Background:**

High dose ionizing radiation (IR) induces potent toxic cell effects mediated by either direct DNA damage or the production of reactive oxygen species (ROS). IR-induced modulations in multiple biological processes have been proposed to be partly regulated by radiosensitive microRNA (miRNA). In order to gain new insights into the role of miRNAs in the regulation of biological processes after IR, we have investigated changes in mRNA and miRNA expression after high dose IR.

**Results:**

IR induced changes in the mRNA and miRNA profiles of human peripheral blood mononuclear cells (PBMCs). When comparing non-irradiated and irradiated samples, we detected a time-dependent increase in differentially expressed mRNAs and miRNAs, with the highest differences detectable 20 hours after exposure. Gene ontology analysis revealed that very early events (up to 4 hours) after irradiation were specifically associated with p53 signaling and apoptotic pathways, whereas a large number of diverse cellular processes were deregulated after 20 hours. Transcription factor analysis of all up-regulated genes confirmed the importance of p53 in the early post-irradiation phase. When analyzing miRNA expression, we found 177 miRNAs that were significantly regulated in the late post-irradiation phase. Integrating miRNA and target gene expression data, we found a significant negative correlation between miRNA-mRNA and identified hepatic leukemia factor (HLF) as a transcription factor down-regulated in the response to IR. These regulated miRNAs and the HLF target genes were involved in modulating radio-responsive pathways, such as apoptosis, the MAKP signaling pathway, endocytosis, and cytokine-cytokine interactions.

**Conclusion:**

Using a large dataset of mRNA and miRNA expression profiles, we describe the interplay of mRNAs and miRNAs in the regulation of gene expression in response to IR at a posttranscriptional level and their involvement in the modulation of radiation-induced biological pathways.

**Electronic supplementary material:**

The online version of this article (doi:10.1186/1471-2164-15-814) contains supplementary material, which is available to authorized users.

## Background

Exposure of living cells to ionizing radiation (IR) results in the generation of free radicals and reactive oxygen species (ROS) [1]. IR has been shown to cause severe cell damage and cell stress, mediated either directly by disturbing DNA integrity or indirectly via formation of ROS and bystander effects [2]. A wide range of different methods have been used to identify cellular responses to IR, ranging from the detection of chromosomal changes and cell viability assays to transcriptional profiling using gene expression array techniques [3–9]. The latter has acquired growing attention in the scientific community because it enables researchers to gain an excellent overview of molecular mechanisms that are altered in response to IR.

The magnitude of the radiation dose [3, 5, 10] and the type of radiation influence gene expression differently [11]. High-dose radiation (>2 Gy) is associated with increased DNA lesion complexity , such as genotoxic stress responses including DNA damage sensing and altered repair mechanisms, as well as immunological alterations [3]. In contrast, the complex mechanisms underlying the altered biological processes after high dose radiation have still not been elucidated completely.

MicroRNAs (miRNAs) are short non-coding RNAs (21 to 23 nucleotides long) that regulate approximately 30-60% of all protein coding genes via imprecise binding of bases with complementary sequences, usually on the 3’ end of the target mRNA [12]. More than 1800 miRNAs have been discovered [13]. miRNAs mediate translational repression by forming RNA-induced silencing complexes and targeting mRNAs by either inhibiting translation or initiating mRNA degradation [14]. A single miRNA can target a sequence of up to thousands of genes. miRNA target genes can be identified using bioinformatics algorithms. These programs mainly determine potential binding sites in 3’UTRs, called seed sequences, which consist of complementary base pairs and are recognized by miRNA base-pairing [15]. In recent years, the accuracy and reliability of computer programs predicting miRNA targets has greatly improved, and novel strategies have been developed for predicting miRNA targets that take into account that miRNAs and their target genes are co-regulated by common transcription factors [15]. Furthermore, bioinformatics databases providing functional information on miRNA-targeted genes are growing and provide deeper insight into the involvement of mRNAs and miRNAs in biological processes at the posttranscriptional level.

Thus, the most valuable information from miRNA profiling can be acquired by comparing the mRNA and miRNA expression profiles of the same biological samples. No paired miRNA-mRNA data from human PBMCs after IR have been published thus far [16–19], and a precise analysis of negatively correlated miRNA-mRNA pairs has been hindered by a lack of paired mRNA transcripts.

In order to improve the biological validity of the data, we analyzed parallel mRNA and miRNA whole genome expression profiles from the same biological samples after exposure to high dose gamma radiation. We used up-to-date bioinformatics analysis to predict IR-inducible expression of transcription factors for miRNAs and mRNAs, as well as miRNA target genes, and the interplay between miRNAs and mRNAs in the regulation of biological pathways. We identified strong alterations in gene and miRNA expression in irradiated versus non-irradiated cells and show that transcription factor p53 and its downstream effector proteins play a central role in the biological response to IR.

## Methods

### Ethics statement

This study was approved by the ethics committee of the Medical University of Vienna (ethics committee vote number: 1236;2013) and conducted according to the principles of the Helsinki Declaration and Good Clinical Practice. Written informed consent was obtained from all participants. Exclusion criteria were any treatment with immunomodulatory medication during the past 4 weeks, any sings of acute infection, pregnancy, and age < 18 years or > 80 years.

### Cell separation and irradiation

Human peripheral blood mononuclear cells (PBMCs) were obtained from four healthy male volunteers by venous blood draw. Cells were separated by Ficoll-Paque (GE Healthcare Bio-Sciences AB, Sweden) density gradient centrifugation. Heparinized anticoagulated blood specimens were processed immediately after venipuncture, diluted 1:2 in Hanks balanced salt solution (HBBS, Lonza, Basel, Switzerland), and transferred carefully to 50 ml tubes containing Ficoll‒Paque solution (GE Healthcare Bio‒Sciences AB, Sweden). The tubes were centrifuged for 15 minutes at 800 g at room temperature without braking and buffy coats with mononuclear cells obtained. Cells were washed in HBSS and resuspended in CellGro serum-free medium (CellGenix, Freiburg, Germany; 25 × 10^6^ cells/ml). Cell concentrations were determined on a Sysmex automated cell counter (Sysmex Inc., USA). PBMCs from the four donors were γ-irradiated with 60 Gray (Gy) of Caesium-137 irradiation. For each experiment, irradiated and non-irradiated PBMCs from the same donor were incubated for 2, 4, and 20 hours.

### Total RNA isolation

At the end of the incubation and immediately after PBMC separation (0 h), total RNA was isolated from approximately 25 × 10^6^ irradiated or non-irradiated PBMCs using Trizol® Reagent (Invitrogen, Carlsbad, CA) according to the manufacturer’s instructions. Total RNA was quantified using a NanoDrop-1000 spectrophotometer (Peglab, Erlangen, Germany) and RNA quality monitored by an Agilent 2100 Bioanalyzer (Agilent, Böblingen, Germany). All RNA samples used in further steps had an RNA integrity score between 5.7 and 10. A total of 28 samples were generated from the four different donors .

### mRNA and microRNA microarray hybridization

mRNA and miRNA expression profiles were obtained for the same samples using the Agilent Whole Human Genome Oligo Microarray (8 × 60K; G4851A; #028004; Agilent Technologies), which detects 27,958 target Entrez gene mRNAs and 7,419 lincRNAs. Briefly, 600 ng Cy3-labeled fragmented cRNA was hybridized overnight to an Agilent whole human genome oligo microarray, washed twice, blocked, and scanned using Agilent’s microarray scanner.

Agilent Human miRNA Microarray Kit (8×60K; G4872A; #031181) based on miRBase release 16.0 and detecting 1,205 human miRNAs was used to analyze miRNA expression. Briefly, 100 ng total RNA was labeled and hybridized to an Agilent human miRNA microarray overnight at 55°C. The microarray was washed twice and fluorescence detected using Agilent’s Microarray Scanner System. Agilent’s Feature Extraction software was used for image analysis. Microarray gene analysis was performed by Milteny (Milteny Biotec GmBH, Germany).

Both mRNA and miRNA data were generated according to the MIAME guidelines [20] and are available on the Gene Expression Omnibus (GEO) website (http://www.ncbi.nlm.nih.gov/geo/) under accession number GEO: GSE55955.

### Statistical analysis of gene expression data

Background-corrected fluorescence intensity values were imported into GeneSpring v.11, log2-transformed, and then normalized by quantile normalization. The mean values of identical replicate probes on each chip were calculated by GeneSpring. The Agilent whole human genome mRNA chip uses different probes for some transcripts. For statistical analysis these transcripts were treated as distinct transcripts, whereas functional analysis was executed with summarized values for different probes of the same gene. A filtering step was applied in order to reduce the number of multiple hypotheses. Only genes for which at least 100% of the values in one of the two conditions (irradiated vs. non-irradiated) were above the 40th percentile of the average expression of all samples were included in the final analysis. The threshold of 40% was chosen because approximately 30-60% of all human genes are expressed [21, 22].

### Statistical analysis of miRNA expression data

The miRNA data were processed as described above for mRNA data. “Filter on Flags” was used as a filtering step for miRNA samples. Flags were attributes that denote the quality of the entities. These values were generated based on the feature quality on the chip. Genes that were given a low significant attribute in the data file were marked as “Absent” and high significant values were marked as “Present”. We used the default settings for GeneSpring in the filtering process, obtaining a total of 241 available human miRNAs. Differentially expressed mRNAs and miRNAs were identified by paired t-tests in GeneSpring. The resulting p-values were corrected for multiplicity by applying Benjamini-Hochberg adjustment to all p-values calculated for a time point with a false discovery rate (FDR) < 5% [23]. Genes with an adjusted p-value <0.05 were considered significant.

### Functional analysis of radiation-responsive genes

To gain information on the significantly expressed genes, we functionally categorized them using the WEB-based Gene Stet Analysis Toolkit (WebGestalt) database [24]. This web-based analysis tool enables the detection of enrichment of gene ontology (GO) terms in a set of genes and uses the Kyoto Encyclopedia of Genes and Genomes (KEGG) annotation to assign pathways that are significantly affected. We used the whole human genome as a reference set for enrichment analysis and applied the Benjamini-Hochberg method for multiple testing with a significance level of p ≤ 0.05 and FDR < 5%.

### Hierarchic clustering

GeneSpring software was used for hierarchic clustering of the miRNA and mRNA expression data. A Euclidean distance metric and complete average-linkage clustering was used for hierarchic clustering.

### Visualization of protein-protein interactions

To visualize known and predicted protein-protein interactions, the web-based database STRING v9.1 (Search Tool for the Retrieval of Interacting Genes/Proteins) was used [25].

### Sylamer analysis

The Sylamer program was used to predict miRNA binding site differences in the 3’UTRs of differentially expressed miRNAs in human PBMCs 20 hours after irradiation [26]. A total of 1,018 up-regulated genes and 1,285 down-regulated genes with a fold change >1.0 in irradiated samples and ordered by relative expression were used for analysis.

### Identification of miRNA target genes and negative-correlation analysis of miRNA and mRNA expression data

The Magia^2^ web tool was used to identify the targets of miRNAs significantly differentially expressed in irradiated PBMCs [27]. Because of the small number of samples, we used the non-parametric Spearman correlation coefficient to estimate the degree of negative correlation (e.g., up-regulated miRNA and down-regulated mRNA target). The TargetScan algorithm was used to predict the biological targets of miRNAs with a stringency of 0.7. Genes with a Spearman correlation coefficient < −0.7 were used to identify enriched biological processes and pathways using the WebGestalt tool.

### Transcription factor binding site analysis

The web-based platform oPOSSUM3.0 (http://opossum.cisreg.ca/oPOSSUM3) was used for transcription factor binding site analysis [28]. oPOSSUM 3.0 detects known transcription factor binding sites in the promoter sequences of co-expressed genes in order to evaluate whether a transcription factor binding site is enriched within the gene set [29]. Upstream sequences (2000 bp) of up-regulated genes were analyzed using the default parameters in oPOSSUM 3.0 Sequence-based Single Site Analysis (SSA). We interrogated the oPOSSUM database for SSA analysis on two sets of genes corresponding to all up-regulated genes 2 or 4 hours after irradiation, and for the highest 1000 up-regulated transcripts 20 hours after radiation.

### Quantitative reverse transcriptase PCR (qPCR) analysis of mRNA

To validate the data on IR-induced genes, changes in the expression of selected mRNAs were evaluated by qPCR. cDNA were transcribed using the IScriptc DNA synthesis kit (BioRad, Hercules, USA) as indicated in the instruction manual. Amplified PCR products were unstitched on a 1.5% agarose gel and stained with GelRed (Biotium, Hayward, USA). DNA Molecular Weight Marker VI (Roche Applied Science, Penzberg, Germany) was used as a reference marker. mRNA expression was quantified by qPCR with Light Cycler Fast Start DNA Master SYBR Green I (Roche Applied Science, Penzberg, Germany) according to the manufacturer’s protocol. The primer pairs were designed as described previously [30] and synthesized by Microsynth AG (Vienna, Austria). The primer sequences are provided in the supplementary material (Additional file 1). The specificity of the PCR products was confirmed by sequencing. The relative expression of target genes was compared to the housekeeping gene beta-2-microglobulin using a formula described by Pfaff et al. [31]. The efficiencies of the primer pairs were determined as described previously [30].

### qPCR analysis of miRNA

The miRNA expression analysis was validated using the TaqMan® MicroRNA Assay Kit (Applied Biosystems, Foster City, CA). Briefly, each RT reaction contained 10 ng of total purified RNA, 5× stem-loop RT primer, 1× RT buffer, 0.25 mM of each dNTP, 50 U MultiScribe™ reverse transcriptase, and 3.8 U RNase inhibitor. The reactions were incubated for 30 min at 16°C, 30 min at 42°C, 5 min at 85°C, and then held at 4°C. The resulting cDNA was amplified quantitatively using LightCycler® Probes Master Mix and Taqman microRNA assays for miR-99b*, miR-887, miR-4299, and miR-RNU44 as an endogenous control. The relative expression levels between samples were calculated as described above.

### FACS analysis

The induction of apoptosis was measured by annexin V-fluorescein/propidium iodide (FITC/PI) co-staining (Becton Dickinson, Franklin Lakes, NJ, USA) using a flow cytometer as described previously [32].

### Apoptosis membrane array and ELISA analysis

An apoptosis antibody membrane-array (Proteom Profiler Arrays, R&D Systems, Minneapolis USA) was used to detect the relative protein concentrations of 35 apoptosis-related proteins. Lysates of irradiated and non-irradiated PBMCs from two donors (cell count 10 × 10^6^ cells) 20 hours after exposure were used according to the manufacturer’s instructions. The dot intensities of apoptosis membrane arrays for semi-quantitative analysis were quantified by volume densitometry using BioRad Image Lab software. Supernatant levels of IL-16 secreted by irradiated and non-irradiated PBMCs at 2, 4, and 20 hours were measured by a commercially available enzyme-linked immunosorbent assay (ELISA, Duoset, R&D Systems, Minneapolis, USA).

### Immunoblot analysis

For the preparation of whole cell lysates, PBMCs were lysed in SDS–PAGE loading buffer, sonicated, centrifuged, and denatured before loading. The protein content of 10^6^ cells 20 hours after irradiation or 10^6^ non-irradiated cells was used for Western blotting. SDS–PAGE was performed on 8–18% gradient gels (GE Amersham Pharmacia Biotech, Uppsala, Sweden). The proteins were then electro-transferred onto nitrocellulose membranes (Bio-Rad, Hercules, CA, USA) and immunodetected using primary antibodies against p53 (1 μg/ml; Abcam, Cambridge, UK), p21(1 μg/ml; Abcam), SP1 (2 μg/ml; New England Biolabs, Beverly, MA, USA), ZFX (1 μg/ml; New England Biolabs), CRTR1 (also known as TFCP2L1; 2 μg/ml; Abcam), HLF (2 μg/ml; Abcam), and KLF4 (1 μg/ml; Abcam). Glyceraldehyde-3-phosphate dehydrogenase (GAPDH) served as the housekeeping protein (0.2 μg/ml; Biogenesis, Poole, UK). Reaction products were detected by chemiluminescence with the Chemi Glow reagent (Biozyme Laboratories Limited, South Wales, UK) according to the manufacturer’s instructions.

### Transmission electron microscopy (TEM)

Irradiated and non-irradiated PBMCs (20 hours after exposure) were dehydrated in a graded ethanol series (50%, 70%, 90%, 96%, and twice in 100%) and embedded in Epon (Serva, Heidelberg, Germany). Ultrathin sections (80–100 nm) were cut using an UltraCut-UCT ultramicrotome (Leica Inc., Vienna, Austria), transferred to copper grids, and viewed either unstained or stained with 1% uranyl acetate and 5% lead citrate (Merck, Darmstadt, Germany) using an EM-900 TEM (Carl Zeiss, Oberkochen, Germany) at an acceleration voltage of 50 kV. Digital images were recorded using a wide-angle dual speed CCD camera (Albert Tröndle, Dünzelbach, Moorenweis, Germany).

### Statistical analysis

Statistical analysis was performed using GraphPad Prism4 software (GraphPad Software, La Jolla, CA, USA). Comparisons between the two groups at a given time point were tested by a paired t-test. Data are expressed as mean ± standard deviation (SD) or displayed as box plots. A two-sided corrected p-value <0.05 was considered significant.

## Results

### Ionizing radiation induces apoptosis in human PBMCs

**Figure 1:**
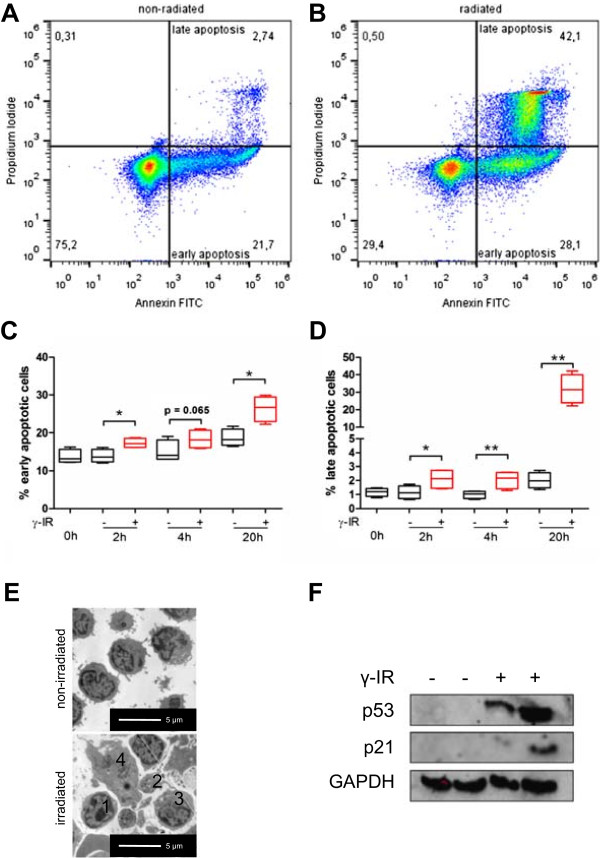
**Ionizing radiation induces apoptosis in PBMCs.** To detect the percentage of early and late phase apoptotic cells, the cells were stained with annexin V and propidium iodide 2, 4, and 20 hours after exposure to IR. FACS analyses of non-irradiated **(A)** and irradiated **(B)** PBMCs 20 hours after exposure are shown. Viable cells are annexin V and propidium iodide-negative (lower left quadrant), whereas early apoptotic cells are annexin V-positive and propidium iodide-negative (lower right quadrant) and late apoptotic cells are double-positive (upper right quadrant). **(C, D)** Quantitative analysis of the FACS data 2, 4, and 20 hours after irradiation is shown. **(E)** Representative electron microscope picture of non-irradiated (upper panel) and irradiated (lower panel) PBMCs 20 hours after exposure. Irradiated PBMCs exhibited morphological signs of late apoptosis, such as chromatin condensation in the nucleus (1), free apoptotic bodies (2), vesicle formation (3), and dissolution of the cell membrane (4). **(F)** A representative immunoblot of p53 and p21 in cell lysates from 10 × 10^6^ PBMCs 20 hours after exposure to IR. Irradiation up-regulated the protein levels of both p53 and its downstream target p21. *p < 0.05, **p < 0.01; n = 4.

IR is known to initiate apoptosis in different cell types, but the underlying molecular mechanisms have not been fully elucidated. To allow comparisons between our study and others, we evaluated the already established effects of irradiation on human PBMCs. Twenty hours after irradiation, the numbers of early apoptotic PBMCs (annexin V-positive cells) and advanced apoptotic PBMCs (annexin V and propidium iodide-positive cells) were significantly higher in irradiated PBMCs than non-irradiated PBMCs (Figure 1A). Approximately 30% of irradiated PBMCs were in a late apoptotic phase, compared to 2% of non-irradiated PBMCs (Figure 1B). A slight increase in the number of early and late apoptotic PBMCs was detectable as early as 2 hours after irradiation (Figure 1C, D). As shown in Figure 1E by electron microscopy, exposure to IR induced morphological abnormalities in PBMCs, such as nucleolus fragmentation and the presence of apoptotic bodies. As IR is known to induce DNA double strand breaks following activation of the tumor suppressor p53, we performed Western blot analysis to quantify p53 and its downstream target p21. As shown in Figure 1F, irradiation of PBMCs with high dose IR (60 Gy) strongly increased the expression of both p53 and p21.

### Ionizing radiation induces the expression of apoptosis-related proteins

**Figure 2:**
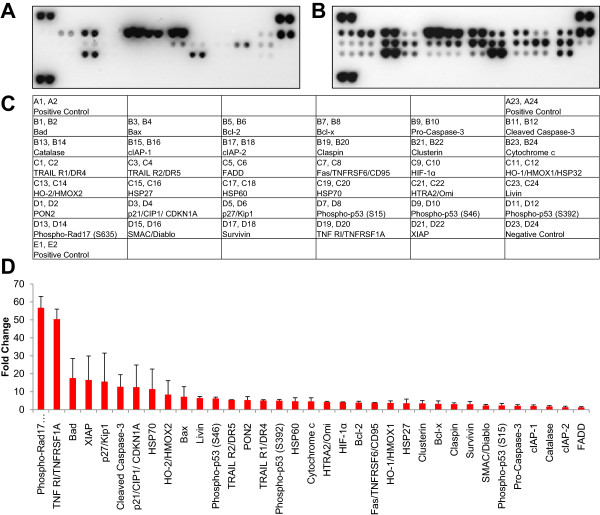
**Proteins involved in executing apoptosis are up-regulated in response to ionizing radiation.** A membrane array detecting 35 apoptosis-related proteins was incubated with cell lysates from 10^6^ non-irradiated **(A)** or irradiated **(B)** human PBMCs 20 hours after exposure. **(C)** The legend for the spotted proteins. **(D)** The average pixel intensities of two independent experiments were averaged and are depicted as the fold change in up-regulation of irradiated PBMCs compared to non-irradiated PBMCs. For relative quantification in **(C)**, an exposure time of 60 seconds was used for proteins with high expression values, and an exposure time of 625 seconds was used for proteins with lower expression values. Data are presented as mean + SD; n = 2.

Next, we analyzed the expression of 35 proteins associated with apoptotic signaling using an antibody membrane array. Cell lysates from irradiated or non-irradiated PBMCs 20 hours after exposure were used for this experiment. A representative picture of the membrane array is shown in Figure 2. All proteins exhibited moderate to high induction in irradiated samples compared to non-irradiated samples (Figure 2D).

**Figure 3:**
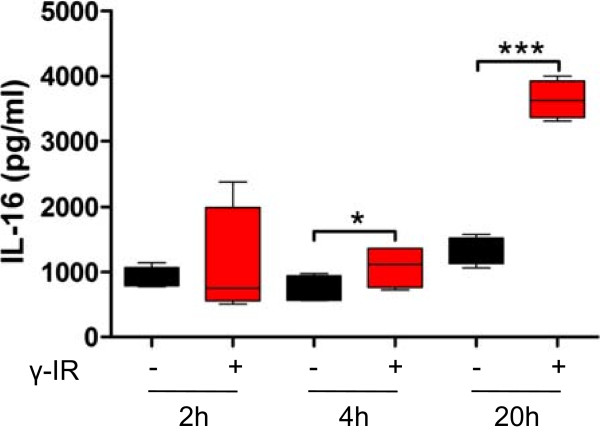
**Caspase-3 activation follows a time-dependent up-regulation after exposure to ionizing radiation.** IL-16 concentrations measured in the supernatant of non-irradiated (black box-plot) and irradiated (red box-plot) PBMCs are shown as a surrogate marker of caspase-3 activation. Supernatants were collected 2, 4, and 20 hours after exposure to IR and IL-16 concentrations measured with ELISA. A time-dependent increment in IL-16 was observed in the supernatant from irradiated PBMCs, with significantly higher levels starting 4 hours after irradiation compared to non-irradiated cells. *p < 0.05, **p < 0.01, ***p < 0.001; n = 4.

We were interested in describing the post-transcriptional regulatory signaling cascades between mRNA and miRNA; therefore, we initially analyzed the regulation and activation of proteins associated with apoptosis and cell cycle in response to IR. As shown in Figure 2, anti-apoptotic proteins such as Bad, BAX, BCL-2, ciap-1, ciap-2, XIAP, and survivin were highly enriched in lysates from irradiated PBMCs. We also detected increased activation of caspase-3, as well as p53 and Rad17, in irradiated cells, which are not regulated at the transcriptional level, but activated by post-translational modifications. Some of the up-regulated proteins, such as p53, p21, p27, and XIAP, are known to induce cell cycle arrest, whereas others exert anti-oxidative functions (e.g., PON2, HO-1, HO-2). We also found heat shock proteins 27, 60, and 70 are up-regulated after irradiation, suggesting an additional impact of molecular chaperones in preserving cell integrity and function. Though the de novo production of proteins takes some time, the activation of pre-synthesized proteins via phosphorylation or cleavage can occur rapidly. Within the caspase cascade, caspase-3 plays a key role in executing apoptosis [33]. Active IL-16 serves as a surrogate marker of caspase-3 activation because pro-IL-16 is cleaved by caspase-3 in monocytes undergoing apoptosis [34]. Therefore, we evaluated the time-dependency of IL-16 concentrations in supernatants from cultures of irradiated and non-irradiated PBMCs as a surrogate marker of caspase-3 activity (Figure 3). Four and 20 hours after irradiation, IL-16 levels were higher in the supernatant from irradiated cells compared to non-irradiated cells (Figure 3), showing a strong correlation with the number of apoptotic cells and the time after irradiation (Figure 1C, D). Caspase-3 activity has been shown to be controlled by miRNA-378 [35]. In this experimental setting miRNA-378 was notably reduced in irradiated cells.

### Matched gene and miRNA expression analysis in irradiated and non-irradiated human PBMCs

Based on the protein data, we obtained the miRNA and mRNA expression profiles of 28 samples obtained from four donors. This dataset includes non-irradiated PBMCs and irradiated PBMCs 2, 4, and 20 hours after exposure to IR, as well as control PBMCs without any cultivation period.

### Time-dependent changes in mRNA expression after irradiation

**Figure 4:**
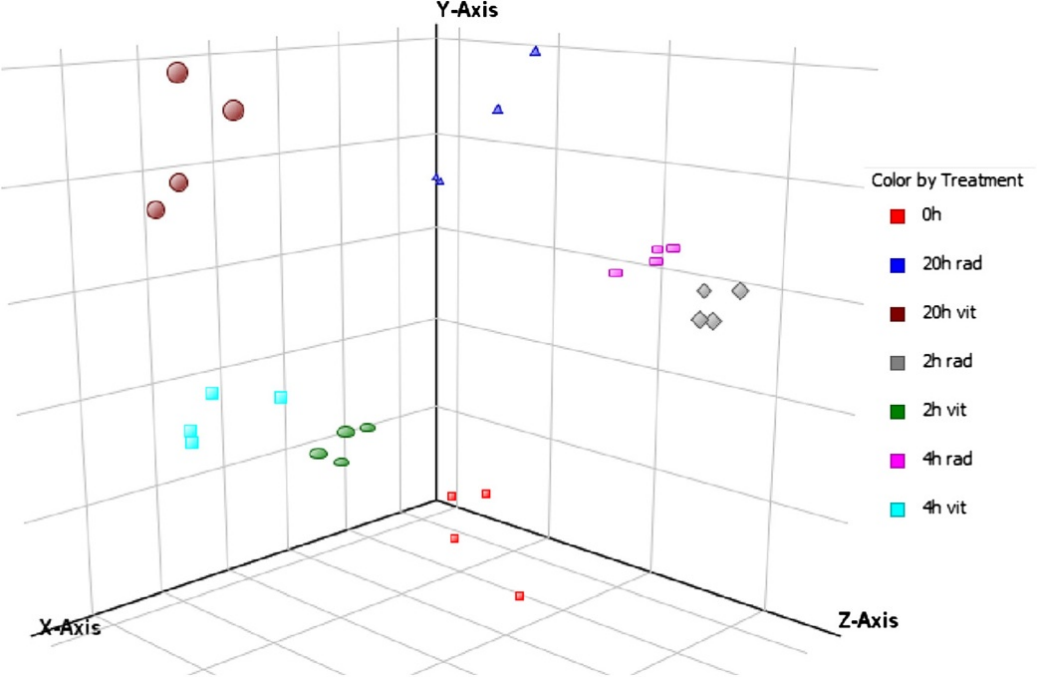
**Principle component analysis (PCA) of mRNA expression data shows differences in response to irradiation and cultivation.** Samples are displayed with respect to the first three components and are colored with respect to radiation and time point. A total of 28 unique experiments were performed and are displayed in the figure. Matched samples of irradiated and non-radiated PBMCs from four different donors at 2, 4, and 20 hours, as well as expression data from PBMCs isolated immediately after venipuncture without any cultivation period, were used for mRNA expression analysis. PCA allows visual identification of data patterns and highlights similarities and differences between samples. PCA was performed using GeneSpring and was based on conditions. All conditions can be clearly separated from each other. Irradiated PBMCs built three distinct clusters on the right side of the figure, displaying relative low X-axis values. In contrast, non-irradiated PBMCs formed three visible clusters, displaying completely distinct X-axis values compared to irradiated PBMCs, whereas the Y-axis and Z-axis values were comparable. The expression data for 0 h PBMCs formed a distinct cluster separate from 2 or 4 hours after cultivation, even without irradiation. 0 h = PBMCs without any cell culture period; rad = irradiated samples; vit = non-irradiated samples.

In order to evaluate time-dependent changes in expression, we compared mRNAs from irradiated PBMCs and non-irradiated PBMCs 2, 4, and 20 hours after exposure to IR. The Agilent Whole Human Genome Microarray (8 × 60K) includes 27,950 mRNAs and 7,419 linc-RNAs. The mRNA expression data is displayed in a principal component analysis (PCA) in Figure 4. PCA depicts expression values as points on a three-dimensional scale. Each dot in the figure represents averaged expression data of one unique sample. A total of 28 dots are visible in Figure 4, corresponding to the 28 experiments performed. Based on this analysis, samples are clustered and separated from each other according to the treatment and time after exposure. Irradiated samples form three distinct clusters are visible on the right side of the figure. Time-dependent irradiated samples increase on the Y-axis and decrease on the Z-axis, whereas the position on the X-axis remains constant. Non-irradiated samples and expression data from PBMCs without any cultivation build a separate cluster on the left side of the figure. Compared to irradiated PBMCs, the non-irradiated PBMCs had higher X-axis values, but the Y-axis and Z-axis values were comparable between matched time points for irradiation and non-irradiated PBMCs. The figure also shows that cultivation of human PBMCs per se alters gene expression, as the 0 h cells can be separated from non-irradiated PBMCs at 2, 4, and 20 hours after cultivation.

**Figure 5:**
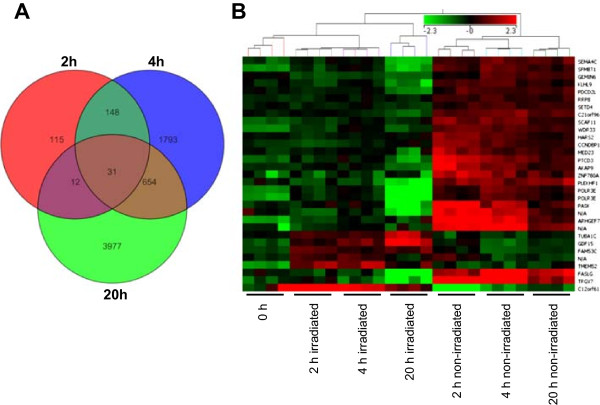
**Ionizing radiation induces alterations in mRNA expression levels. (A)** mRNA changes in irradiated human PBMCs incubated for 2, 4, and 20 hours using the Agilent Whole Human Genome Oligo Microarray (8 × 60 K) are shown. Venn diagram showing the overlap of up- and down-regulated genes with significant changes in expression 2, 4, and 20 hours after irradiation. A time-dependent increase in differentially expressed genes was observed. The greatest alterations in gene expression between irradiated and non-irradiated PBMCs were detected 20 hours after exposure. The inner triangle contains 31 transcripts that were differentially expressed in all samples at all time points. **(B)** Heat map showing the expression values of the 31 transcripts that were differentially expressed at all time points in all donors. The range of expression was from −2.3 (green, down-regulation) to 2.3 (red, up-regulation). Each time point can be clearly discriminated, and irradiated samples can be discriminated from non-irradiated samples. miRNA names are given on the right border of the heat map.

We performed statistical analyses on gene expression data in order to quantify differentially expressed transcripts in irradiated versus non-irradiated PBMCs. Only genes for which at least 100% of the expression values in one of the two conditions were above the 40th percentile were used for statistical analysis. We detected a time-dependent increment of differentially expressed mRNA. Two hours after irradiation, 16,660 transcripts were tested with Student’s T-test; 608 of these transcripts exhibited significant differences, 306 with a fold change > 1.5 (152 up-regulated, 154 down-regulated in irradiated PBMCs). Four hours after irradiation, 17,428 transcripts were tested with Student’s T-test; 4,746 of these transcripts exhibited significant differences, 2,626 with a fold change >1.5 (869 up-regulated, 1,757 down-regulated in irradiated PBMCs). Twenty hours after irradiation, 22,586 transcripts were processed after the filtering step; 5,286 of these transcripts exhibited significant differences, 4,674 with a fold change >1.5 (2,770 up-regulated, 1,904 down-regulated in irradiated PBMCs; Additional file 2). The Venn diagram in Figure 5A shows significantly up- or down-regulated transcripts at each time point and the intersection of the expression of these genes. Separate diagrams for up- and down-regulated genes are shown in Additional file 3. A set of 31 genes was significantly altered at every time point in any sample pair from each donor (Figure 5A). Five genes were up-regulated at 2 hours after irradiation, one gene was down-regulated at 2 hours and up-regulated at 4 and 20 hours, and the remaining 25 transcripts were down-regulated at all time points after irradiation (Additional file 4). Hierarchical clustering of this core set of genes enabled us to discriminate irradiated from non-irradiated samples and aided in further identification of the samples at each time point (Figure 5B and Additional file 5).

### Functional annotation clustering

Functional enrichment analysis was performed to identify pathways and biological processes affected in response to IR. Genes with known biological functions were uploaded into the WebGestalt database and classified according to GO terms and (KEGG pathway categories. Default parameters were used for data analysis. Two hours after irradiation, up-regulated genes attributed to processes such as the p53 signaling pathway, apoptosis, and microtubule-based movement were significantly enriched (Additional file 6). The most enriched pathway 4 hours after irradiation was p53 signaling with 16 associated transcripts and an enrichment ratio of 15.5 (Additional file 7). Up-regulated genes 20 hours after exposure to IR were clustered in a variety of biological processes and pathways, with a predominant induction of metabolic, lysosome, phagosome, and cancer-associated pathways (Additional file 8). The protein-protein interactions of 16 up-regulated transcripts coding for proteins in the p53 signaling pathway 4 hours after irradiation were visualized with STRING (Additional file 9). The biological functions of down-regulated genes 2 and 4 hours after irradiation were associated with nucleic acid metabolic processes, RNA metabolic processes, and gene expression (Additional files 10 and 11). Twenty hours after IR, down-regulated genes were associated with lymphocyte activation, hemopoiesis, and somatic cell DNA recombination (Additional file 12). In order to reduce the number of false-positive transcripts, which were only up-regulated at one time point, we also ran analyses with genes that were significantly different at at least two of the three time points. Using this calculation strategy, we detected comparable results showing an induction of biological processes such as the intrinsic apoptotic signaling pathway, regulation of mitochondrial membrane permeability, and KEGG pathways p53 signaling, Wnt signaling, and ubiquitin-mediated proteolysis (Additional file 13).

### Radio-responsive miRNAs

**Figure 6:**
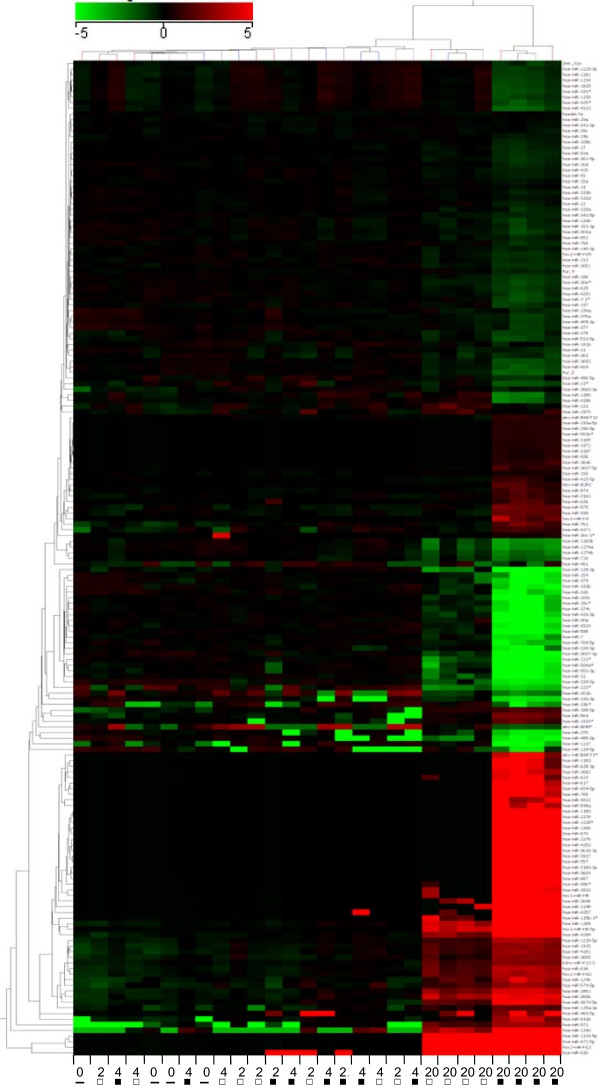
**Ionizing radiation induces alterations in microRNA expression levels.** Differentially expressed miRNAs in irradiated versus non-irradiated PBMCs are shown. The heat map shows 177 miRNAs that were differentially expressed 20 hours after irradiation. Samples obtained 20 hours after irradiation formed a cluster visible on the right side of the figure, whereas prior time points did not cluster according to treatment or time. The range of expression was from −5 (green, down-regulation) to 5 (red, up-regulation). The names of the genes are given on the right border of the heat map. Sample labeling: “2” = 2 hours, “4” = 4 hours, “20” = 20 hours, “▬” = 0 h sample; “□” = non-irradiated sample; “■” = irradiated sample.

As miRNAs are involved in multiple cellular processes and exert regulatory functions, we investigated whether IR induces changes in the miRNA expression levels in irradiated PBMCs. Of the 1350 miRNAs present on the miRNA-array, 241 were considered for further analyses after an initial filtering step. Though we did not identify any significant changes in miRNA expression 2 hours after irradiation, and only seven miRNAs were altered after 4 hours (Additional file 14 Sheet A), 80 miRNAs were up-regulated and 97 down-regulated 20 hours after irradiation (Additional file 14 Sheet B). These 177 miRNAs were clustered according to their expression, showing that miRNA changes were detectable 20 hours after cultivation with up- or down-regulation in irradiated cells compared to other time points (Figure 6).

### miRNA target gene prediction based on mRNA expression with Sylamer analysis

Sylamer analysis is a bioinformatics program that identifies putative miRNA binding sites in the 3’UTR of mRNA based on the nucleotide sequences and calculates whether the predicted targeting miRNAs differ from random expectation in a rank-order list of mRNAs. In general, Sylamer analysis is used to detect alterations in gene expression after miRNA knock-down or in over-expression experiments. We used Sylamer analysis to evaluate whether differentially expressed mRNAs in irradiated PBMCs share an enrichment of specialized miRNA binding sites.

A total of 1,018 up-regulated mRNAs and 1,285 down-regulated mRNAs with a fold change >1.0 were used for analysis. We did not detect enrichment of 3’UTR binding sites for a single miRNA. However, several miRNAs exhibited comparable enrichment of seed sequences in the set of regulated genes. In a further analysis, we evaluated whether we could detect binding sites of known regulated miRNAs in response to IR based on miRNA expression data. We focused on the 10 most differentially expressed genes in irradiated versus non-irradiated PBMCs. In addition, we evaluated the 10 miRNAs with the highest expression values because even small changes in expression could have a large impact on mRNA expression.

Thus, we were able to identify seven up-regulated miRNAs in irradiated PBMCs that exhibited a significant enrichment of seed sequences in the set of down-regulated mRNAs 20 h after irradiation (Table 1). Of these seven miRNAs, four were significantly more highly expressed in irradiated PBMCs and three miRNAs with high absolute ct-values were more highly expressed in irradiated PBMCs, although the difference was not significant. miR-1268 had the highest enrichment score of all detected miRNAs. The corresponding down-regulated target genes of these seven miRNA are given in Additional file 15.

**Table 1:** **Identification of miRNA 3’UTR binding sites in the set of down-regulated mRNAs**

miRNA	Non-irradiated 20 h	Irradiated 20 h	p-value	Target genes	Seed counts
**miR-887**	−0.06	7.83	5.46E-05	76	87
**miR-1306**	−0.06	7.31	4.13E-04	18	18
**miR-1180**	−0.06	6.71	4.25E-04	79	87
**miR-1268**	3.71	6.46	4.93E-03	199	272
**miR-371-5p**	7.07	8.55	ns.	79	87
**miR-630**	6.92	8.28	ns.	383	709

1018 down-regulated mRNAs 20 hours after irradiation with FC > 1.0 were used for Sylamer analysis. p-value: difference between non-irradiated and irradiated PBMCs based on microarray expression data; target genes: number of genes targeted by the miRNA; seed counts: number of unique seeding regions in the given miRNA.

We performed a similar analysis for up-regulated mRNAs in irradiated PBMCs 20 hours after irradiation. Six miRNAs exhibited significant enrichment of 3’UTR binding sites in the set of down-regulated mRNAs (Table 2). Their target genes are given in Additional file 16.

**Table 2:** **Identification of miRNA 3’UTR binding sites in the set of up-regulated mRNAs**

miRNA	Non-irradiated 20 h	Irradiated 20 h	p-value	Target genes	Seed counts
**miR-1237**	−0.04	−4.84	1.83E-03	457	884
**miR-30a**	−0.14	−4.93	1.03E-03	398	796
**miR-598**	−0.51	−4.95	6.18E-04	69	74
**miR-601**	2.89	0.67	ns.	173	212
miR-205*	3.26	2.59	ns.	361	650
**miR-328**	2.24	0.08	ns.	371	633

1285 up-regulated mRNAs 20 hours after irradiation with FC > 1.0 were used for Sylamer analysis. p-value: difference between non-irradiated and irradiated PBMCs based on microarray expression data; target genes: number of genes targeted by the miRNA; seed counts: number of unique seeding regions in the given miRNA.

The same analysis was performed using bioinformatics algorithm cWords (http://servers.binf.ku.dk/cwords/) and revealed comparable results.

### Integrated miRNA-mRNA correlation analysis based on Magia^2^

In order to correlate miRNA and mRNA data obtained by microarray analysis of the same biological samples, we performed negative correlation analysis using Magia^2^. Sylamer analysis was performed with mRNA expression exclusively, whereas the Magia^2^ program combines paired miRNA and mRNA data to predict interaction networks. The miRNA and mRNA expression profiles of paired samples were analyzed only in the 20 hour miRNA data set, as the number of significant miRNAs in the 4 hour dataset was too low to obtain reliable results. Negatively correlated miRNA-mRNA pairs with a correlation coefficient < −0.7 were used for further analysis, and transcripts were uploaded into WebGestalt and classified according to GO terms and KEGG pathway enrichment. Down-regulated miRNA and corresponding up-regulated mRNA were found to be involved in endocytosis, regulation of cell communication, regulation of actin cytoskeleton polymerization, and apoptosis, among others (Additional file 17). Up-regulated mRNAs and corresponding miRNAs for apoptosis and endocytosis are given in Additional files 18 and 19.

Up-regulated miRNAs and their corresponding down-regulated target genes were associated with cell cycle, mRNA surveillance pathway, leukocyte proliferation, and regulation of gene expression (Additional file 20). Down-regulated mRNAs and corresponding miRNAs associated with the cell cycle are shown in Additional file 21.

### Identification of over-represented transcription factor binding sites in sets of differentially expressed genes in response to irradiation

**Figure 7:**
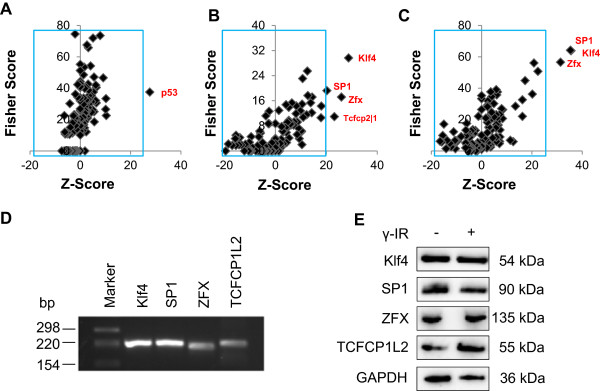
**oPOSSUM analysis of over-represented transcription factor binding sites.** Up-regulated transcripts 2, 4, and 20 hours after irradiation were uploaded to identify over-represented transcription factor binding sites in these sets of genes. Transcription factor binding sites in red highlight transcription factors enriched in the corresponding gene set. **(A)** Two hours after irradiation, p53 was identified as the only putative transcription factor. **(B)** Four hours after IR, Klf4, SP1, Zfx, and TCFCP2L1 were identified as possible transcription factors. **(C)** Twenty hours after irradiation Klf4, SP1, and Zfx were identified as potential transcription factors in irradiated samples. **(D)** PCR validation of Klf4, SP1, Zfx, TCFCP1L2, and HLF in irradiated PBMCs 20 hours after radiation confirmed the expression of these genes at the mRNA level. **(E)** Immunoblotting showed the presence of the predicted transcription factor proteins (Klf4, SP1, Zfx, TCFCP1L2) in both irradiated and non-irradiated samples 20 hours after irradiation. One representative experiment of two is shown. GAPDH served as a control.

To identify transcription factors involved in radio-responsive mRNA changes, oPOSSUM3 was used to identify over-represented transcription factor binding sites in the promoter sets of differentially expressed genes 2, 4, and 20 hours after exposure to IR. This analysis revealed that p53 binding sites were specifically enriched in the promoter regions of genes 2 hours after irradiation (Figure 7A and Additional file 22 Sheet A), suggesting an important role of p53, especially shortly after irradiation. In contrast, p53 could not be identified as a regulatory transcription factor 4 and 20 hours after exposure. At these time points, Kruppel-like factor 4 (Klf4), zinc finger protein X-linked (Zfx), Sp1 transcription factor (SP1), and transcription factor CP2-like 1 (TFCP2L1) binding sites were significantly enriched within the promoter regions (Figure 7B, C). A total of 368 and 548 genes contained binding sites for Klf4 within their promoters 4 and 20 hours after irradiation, respectively (Additional file 22 Sheets B and C).

To further investigate the putative involvement of the predicted transcription factors in the modulation of IR-induced alterations in gene expression, we performed PCR analysis of these transcription factors. Firstly, we were able to show that Klf4, SP1, Zfx, and TFCP2L1 were detectable at the mRNA level (Figure 7D). Secondly, we analyzed whether Klf4, SP1, Zfx, and TFCP2L1 are differentially expressed at the protein level. As shown in Figure 7E, all predicted transcription factors were present at the protein level in cell lysates from irradiated and non-irradiated PBMCs 20 hours after irradiation.

### Integrated miRNA-mRNA analysis suggests hepatic leukemia factor involvement in IR-induced transcriptional regulation

**Figure 8:**
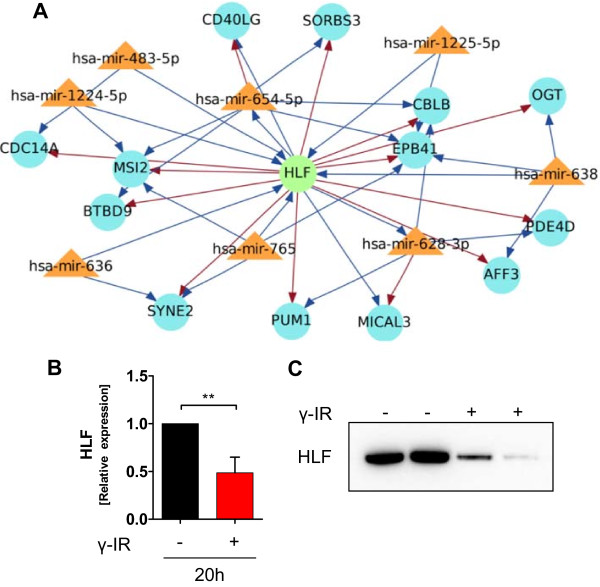
**Regulatory network of hepatic leukemia factor (HLF) displaying the interplay among miRNAs, HLF, and mRNAs. (A)** The network was constructed using MAGIA^2^, which incorporates positive and negative correlations between expression profiles of possibly interacting miRNAs, transcription factors, and genes according to their predictions. These miRNAs were up-regulated in irradiated cells 20 hours after exposure, whereas their target mRNAs were down-regulated. mRNAs, transcription factors, and miRNAs are shown as light blue circles, green circles, and orange triangles, respectively, and positive and negative correlations are shown as red and blue arrows, respectively. **(B)** qPCR of HLF shows a significant reduction in HLF expression 20 hours after IR exposure, n = 3. **(C)** Cells were harvested 20 hours after irradiation and the HLF level assessed by immunoblotting. IR significantly reduced the HLF protein level compared to non-irradiated samples.

Next, we sought to determine whether a transcription factor could be involved in regulatory events affecting both miRNA and mRNA using the Magia^2^ database. Within the miRNA-mRNA correlation analysis, the transcription factor hepatic leukemia factor (HLF) occupies a central position. A representative interactive network with HLF is shown in Figure 8A. On one hand, HLF is the target of several up-regulated miRNAs in irradiated PBMCs, but on the other hand it is a transcription factor for many genes found to be down-regulated in irradiated samples. Based on microarray data, HLF itself should be down-regulated in irradiated PBMCs. qPCR showed that HLF expression levels were significantly lower in irradiated PBMCs compared to non-irradiated PBMCs 20 hours after irradiation (Figure 8B). We performed immunoblot analysis in order to determine if HLF is also regulated at the protein level in response to IR. As shown in Figure 8C, HLF protein levels were lower in irradiated PBMCs compared to non-irradiated PBMCs, which is in line with the data obtained from the miRNA-mRNA expression profiles.

To determine the effect of reduced HLF levels we evaluated the biological function of genes containing a promoter sequence for HLF. These HLF targeted genes were significantly enriched in the regulation of endocytosis pathways (CBLB, IQSEC3), cytoskeleton organization processes, and actin cytoskeleton organization (SORBS3, MICAL, EPB41, OGT).

In conclusion, we identified HLF as a potential transcription factor involved in the regulation of both miRNA and mRNA transcription based on the over-representation of promoter binding sites.

### qPCR validation study

**Figure 9:**
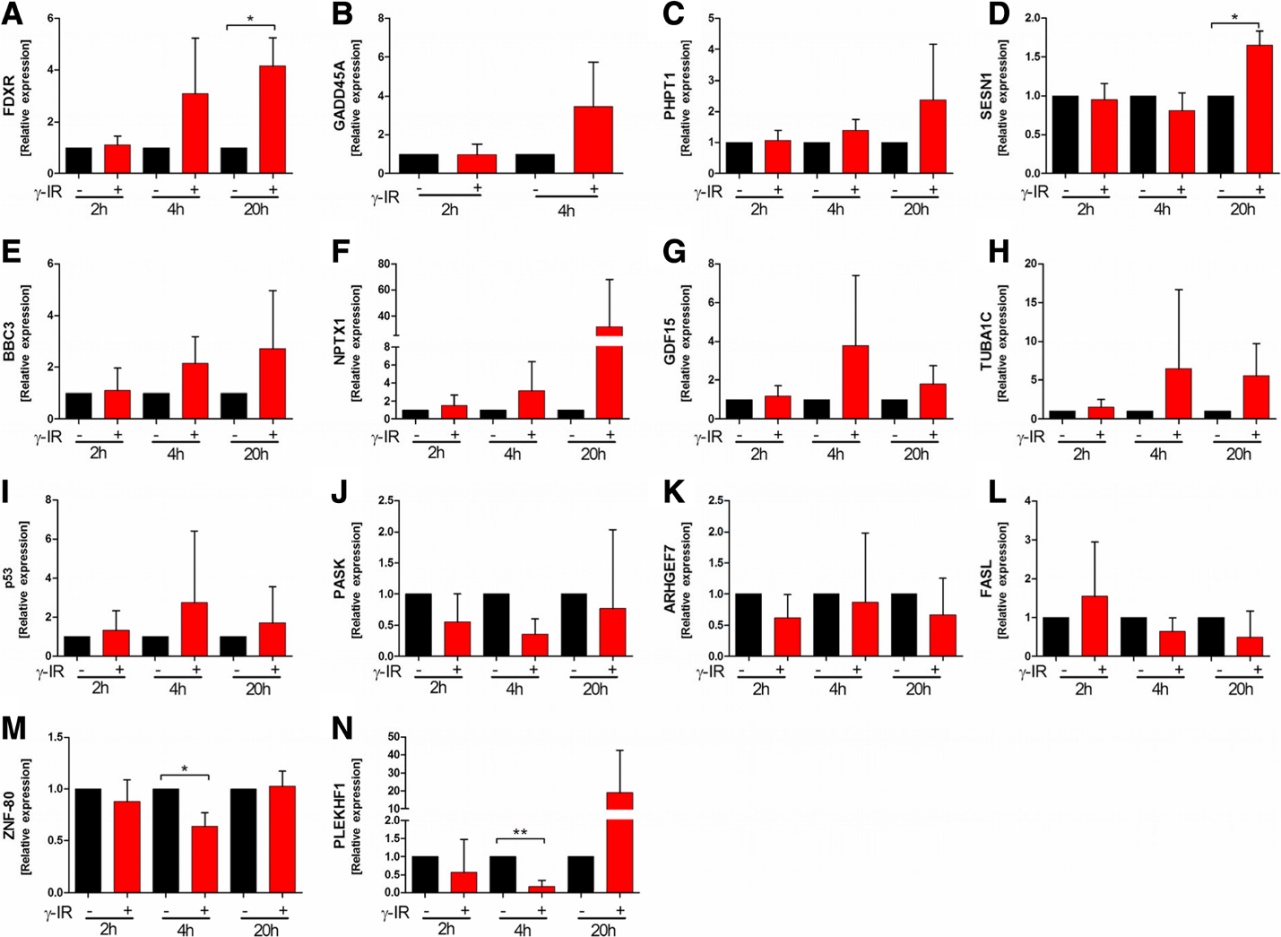
**Validation of the microarray data by qPCR.** Irradiated and non-irradiated human PBMCs were cultured for 2, 4, or 20 hours prior to RNA isolation. Expression values were normalized relative to values in non-irradiated PBMCs. Relative gene expression levels were calculated based on the mean value from four samples using the comparative Ct method. Samples were normalized to the housekeeping gene B2M. Data are presented as mean + SD. Genes in panels **A-I** were up-regulated on the microarray, whereas genes in panels **J-N** were down-regulated in irradiated samples compared to non-irradiated samples. The qPCR data generally supported the microarray data. *p < 0.05, paired T-test; n = 4.

**Figure 10:**
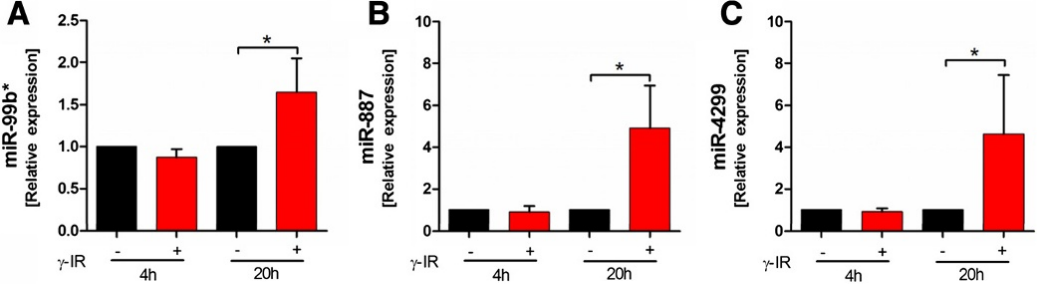
**Validation of the miRNA data by qPCR.** qPCR revealed a strong correlation between the microarray and PCR data. Expression values were normalized relative to values in non-irradiated PBMCs. The expression of miR-99b* **(A)**, miR-887 **(B)**, and miR-4299 **(C)**, was significantly higher in irradiated PBMCs compared to non-irradiated PBMCs 20 hours after irradiation. Relative gene expression levels were calculated based on the mean value of four samples using the comparative Ct method. RNU44 served as internal reverence miRNA. Data are presented as the mean + SD. *p < 0.05, paired T-test; n = 4.

To validate the results from the microarray experiments, 14 genes were chosen for qPCR. Among these genes, nine were up-regulated (Figure 9A-I) and five down-regulated in irradiated PBMCs (Figure 9J-N). We chose a panel of five irradiation-responsive genes (FDXR, CDKN1A, SESN1, BBC3, PHPT1) previously evaluated by Amundson et al., as well as nine genes based on our data analysis [7]. For all genes analyzed, the expression profiles agreed with the microarray data, though the sample size was too small to reach significance in all samples. These data suggest that the results of the microarray analysis were reliable indicators of overall alterations in gene expression. In addition, three miRNAs were chosen for qPCR validation and miRNA expression 4 and 20 hours after irradiation. All miRNAs were significantly up-regulated in irradiated PBMCs compared to non-irradiated PBMCs 20 hours after exposure, whereas no significant changes were observed 4 hours after exposure to IR, which was in line with the microarray data (Figure 10). The overall fold change in expression was comparable for miR-887 and miR-4299, but the fold change for miR-99b* was lower in qPCR than in the chip data.

## Discussion

In this study we showed that high dose IR induces alterations in gene and miRNA expression in human PBMCs, with the greatest changes 20 hours after exposure. We identified p53 and its downstream target proteins as major key players in the modulation of IR-induced biological processes. In addition, miRNA target prediction analysis revealed their involvement in post-transcriptional regulation of gene expression in response to IR.

In the first part of this study we focused on morphological and post-translational changes induced by IR in human PBMCs. We showed that, even 2 hours after irradiation, apoptotic markers, such as phosphatidylcholine, were detectable on the outer side of the plasma membrane and that these markers increased in a time-dependent manner. In recent publications we have reported that, in response to IR, PBMCs are able to release paracrine factors that exert immune modulatory functions and have presented evidence that IR-induced apoptosis is responsible for the release of these paracrine mediators [32, 36–40]. Here, we have identified several up-regulated proteins associated with irradiated apoptotic PBMCs. These proteins were either activated in response to radiation through phosphorylation or cleavage (e.g., phopho-rad17 or cleaved caspase-3) or synthesized de novo (e.g., BAD, XIAP, p21, BAX). Notably, not only proteins synthesized de novo, but also biological activation of stored pro-enzymes, contributed to IR-induced cell responses. Caspases are an example of such important pro-enzymes that are rapidly activated upon cell injury, ultimately leading to apoptosis [41]. As the biological activity of caspases is not reflected at the level of gene expression, we investigated the effects of IR on caspase-3 activation in our experimental setting by measuring IL-16 concentrations in the cell culture supernatant. Biologically inactive pro-IL-16 is stored in the cell nucleus. Caspase 3-mediated cleavage of pro-IL-16 releases mature IL-16 into the extracellular space [34]. Therefore, IL-16 has been proposed as a surrogate marker of caspase 3-mediated apoptosis in PBMCs [34].

In the second part of this study we investigated the extent to which IR induces changes in mRNA and miRNA expression, whether miRNAs are involved in IR-induced gene expression alterations, and whether these gene expression modifications are also reflected in corresponding protein concentrations. Several studies have highlighted that the time after irradiation is an important variable when measuring radiation-induced changes in gene expression [4, 5]. We chose 2, 4, and 20 hours based on previous publications, which have shown a time-dependent increase in the variation in mRNA and miRNA expression, with a peak 20 to 24 hours after exposure [4]. Albrecht and colleges showed that in vitro cultivation of human cells itself alters gene expression over time [42]. To exclude artificial changes in expression due to different culture periods, we compared the mRNA and miRNA expression profiles of PBMCs in matched irradiated and non-irradiated samples incubated for the same time periods. Furthermore, we determined gene and miRNA expression in naïve PBMCs that were not cultured at all. In agreement with Albrecht et al., we observed that culturing human PBMCs induces transcriptome alterations as a function of time, independent of radiation treatment. Unspecific cell activation upon contact with foreign surfaces and unfavorable culture conditions for some sub-populations may be responsible for this observation [42].

IR-induced transcriptional changes 2 hours after exposure included pathways involved in apoptosis, signaling cascades, cell cycle regulation, and cancer formation. Surprisingly, early response genes were also significantly associated with protein polymerization, microtubule-based movement, de novo post-translational protein folding, and ribonucleotide binding. The tumor suppressor gene p53 was specifically up-regulated 2 hours after exposure. The p53 response to IR has been described extensively. More than 100 p53 target genes involved in regulation of the cell cycle, transcription, cell death, and DNA repair have been discovered [43]. The involvement of p53 has been described primarily for high dose radiation [44], whereas its role in the response to low doses remains controversial [45]. Our results are in line with those of others that have shown that IR induces rapid expression of p53, and that other genes associated with IR damage are not detectable at an early time point. In addition to p53, cytochrome c, a second known IR responsive gene, was up-regulated 2 hours after exposure. Cytochrome c activates the caspase cascade and is a key player in initiating apoptosis. Therefore, these data indicate that, as early as 2 hours after IR injury, two key mediators of DNA repair and apoptosis were up-regulated, whereas other previously described radiation-induced genes were not detectable. These observations seem to be explicable by the fact that high dose radiation induces severe double strand breaks [46], as well as clustered DNA lesions [47], which are powerful inducers of p53 accumulation, activation, and expression. In addition to the described functions of p53 in response to high dose radiation, recent studies in a model of ex vivo irradiated human skin indicate that p53 also plays important roles during low dose irradiation. For example, in low dose radiation injury p53 seems to promote cell survival via activation of cell protective pathways, DNA repair, and cell cycle arrest, whereas in high radiation injury cell death executer functions are predominantly induced by p53 [42].

Functional clustering and analysis of genes significantly up-regulated compared to control samples 4 hours after irradiation revealed strong enhancement of apoptosis-related proteins. We were able to identify several novel pro-apoptotic transcripts up-regulated at this time point but not at 2 hours: SESN2, DDB2, TNFRSF10B, P21, CCND1, MDM2, PMAIP1, PTEN, GADD45B, CCND3, BAX, BBC3, ZMAT3, and SFN. These gene expression data are in line with our protein data; we observed higher concentrations of these proteins in irradiated PBMCs 20 hours after exposure. Apparently, between 2 and 4 hours after irradiation the transcriptional machinery of the IR-induced damage response is activated by p53, consequently up-regulating several transcripts. The importance of p53 was also confirmed by transcription factor binding site analysis. Two hours after exposure, p53 was the only enriched transcription factor. Interestingly, the transcription factor Klf4, a direct downstream target of p53, had binding sites in the promoter region of 368 and 548 up-regulated transcripts 4 and 20 hours after exposure, respectively. Transcription factor binding sites for p53 were not enriched in the gene set 4 and 20 hours after irradiation. The tumor suppressor Klf4 activates p21 transcription and inhibits cell proliferation [48]. These data suggest that rapid activation of p53 results in up-regulation of Klf4, which amplifies the p53-mediated response to IR.

We identified enrichment of transcription factor binding sites within p53 and Klf4 for SP1, Zfx, and TFCP2L1 in the set of up-regulated mRNAs 4 and 20 hours after irradiation. We validated these data using PCR to show that these transcription factors were expressed in irradiated PBMCs, and immunoblotting confirmed their presence at the protein level, though we could not detect an induction of total protein content in response to IR. Translational modifications, such as phosphorylation or adenylylation, may also be involved in modulating the activity of Klf4, SP1, Zfx, and TFCP2L1; therefore, total protein content does not perfectly correspond to biological activity. Phosphorylation of p53 enhances Klf4 binding, increases Klf4 activity [49], and promotes cell cycle arrest via induction of p21 and p27 [50]. In addition, phosphorylation of SP1 and p53 promotes their regulatory activity and p21 activation [51]. Recently, SP1 was shown to be a central modulator of p53-induced apoptosis in humans [52]. Zfx and TFCP2L1 have multiple phosphorylation sites, though to the best of our knowledge no data exists as to whether post-translational modification is important in regulation of their biological function.

When comparing differentially expressed genes 20 hours after exposure to IR to those differentially expressed 2 and 4 hours after irradiation, we found 4,674 differentially expressed genes. Functional clustering revealed that the majority of these transcripts were involved in the regulation of signal transduction, vesicle-mediated transport, the cytoskeleton, and endocytosis. These data support our FACS analysis and EM photographs, which show that the majority of cells are in a late stage of apoptosis at these time points and that proteins associated with vesicle transport and endocytosis contribute to the formation of apoptotic bodies and cell membrane evaginations. Gene expression data revealed that the process of cellular fragmentation is actively controlled by proteins rather than being a spontaneous occurrence [53].

To further elucidate the IR-induced changes in gene expression, we evaluated whether the expression of miRNAs is affected in response to IR. IR altered the miRNA expression profile as a function of time, which was in accordance with data from human PBMCs [4] and normal human fibroblasts [16]. Significant differences became detectable after 4 hours. Twenty hours after exposure to IR, 177 miRNAs were differentially expressed. Only a minority of the radio-responsive miRNAs identified in this study have been reported in previous studies. One reason for this observation may be the high IR dosage used in our study. Furthermore, progress in the field of miRNA research over the last few years has led to a rapidly increasing number of detectable miRNAs. Eighty of the differentially regulated miRNAs were up-regulated and 97 down-regulated. The predominant down-regulation of miRNAs found in this study was in line with the findings of Joly-Tonetti et al., who showed that all modulated miRNAs of proliferating keratinocytes irradiated with 6 Gy were down-regulated [17]. Giardi and colleges observed a similar tendency, showing an excess of down-regulated miRNAs 24 hours after 2 Gy gamma irradiation of human PBMCs [4]. A possible explanation for this phenomenon is that miRNAs can affect cellular radio-sensitivity by targeting radio-protective genes. Over-expression of miR-9, let-7 g [54], miR-100, miR-101, miR-181a, or miR-421 has been shown to enhance the vulnerability of cells to IR-induced injury [55] and direct cells towards an apoptotic pathway. We observed that several miRNAs with known cell survival activity were down-regulated in response to IR, whereas their pro-apoptotic target genes were up-regulated. For example, miR-21, a pro-oncogenic miRNA known to promote cell proliferation and the evasion of apoptosis, was down-regulated in our irradiated PBMCs, but its targets p53, BAX, PTEN, and BCL2 [56] were up-regulated at both the gene and protein levels. A further down-regulated miRNA in response to IR was miR-378. Caspase-3 is a target protein of miR-378, and over-expression of this miRNA has been shown to inhibit apoptosis, whereas inhibition of miR-378 aggravates hypoxia-induced apoptosis [35]. Moreover, members of the miR-30 family regulate apoptosis by controlling mitochondrial fission, suppressing p53 [57] and caspase 3 translation [58], as well as tumor necrosis factor-related apoptosis inducing ligand-mediated apoptosis [59]. In our data set, we detected a significant down-regulation of miR-30d, miR-30e*, and miR-30a 20 hours after irradiation (see Additional file 14). A down-regulation of these miRNAs in irradiated PBMCs can be interpreted as follows: basal expression of these miRNAs is needed to maintain balance in the translation and degradation of genes coding for proteins involved in cell proliferation and apoptosis. Upon cell injury, transcription of pro-apoptotic genes is enhanced and the expression of targeting miRNAs reduced simultaneously. This coupled mechanism may enable cells to rapidly amplify biological responses to stressful events and maintain cellular integrity.

In accordance with other studies [55], we have shown that miRNAs are involved in regulation of the MAPK pathway in response to IR and in the regulation of apoptosis [60]. Other pathways, such as the mRNA surveillance pathway or T-cell receptor signaling pathway, were repressed by miRNAs. Several studies have documented the involvement of the endocytosis and cytoskeleton pathways in response to radiation exposure [61–63], as well as their alteration through miRNAs [60]. We obtained similar results showing the up-regulation of genes (GATA2, SERPINE1, myosin VA, or ubiquitin specific peptidase 33) associated with endocytosis, vesicle-mediated transport, and signal transduction, whereas their corresponding miRNAs were down-regulated (e.g., mir-32, miR- 92a, miR-200c, miR, or miR-301b).

Using a combination of miRNA-mRNA correlation data, we identified HLF as a putative down-regulated transcription factor in response to IR. HLF is targeted by several up-regulated miRNAs, and its gene expression and protein levels were reduced in irradiated PBMCs compared to non-irradiated PBMCs. This reduction may be due to degradation or inhibition of miRNA-mediated mRNA translation. One previous study has shown that ectopic HLF expression attenuates IR-induced cell death by up-regulating anti-apoptotic genes and repressing the transcription of pro-apoptotic genes. These data are in line with our observation of reduced HLF levels in cells undergoing apoptosis. In addition to the involvement of HLF in the inhibition of cell death pathways, several target genes of HLF were down-regulated in irradiated PBMCs. These genes were involved in the regulation of cytoskeletal processes (SYNE2, EPB41, SORBS3), actin filament depolymerization (MICAL3), and endocytosis (CBLB, JQSEC3). We hypothesize that reduced HLF levels facilitate cell death and modulate morphological-cytoskeletal changes in irradiated samples.

This finding is of special interest because cells in the late phase of apoptosis form plasma membrane blebs containing organelles that are strangulated and released into the extracellular space [64]. The process of plasma membrane budding is actively enforced by cells and mediated by actin-myosin interactions [64]. We found 100 up-regulated mRNAs with known molecular functions in cytoskeletal protein binding, with actin and myosin being the most prominent genes. On the other hand, we detected distinct transcripts actively repressed by up-regulation of distinct miRNAs that were induced upon IR. These data support the notion that cellular dissolution is an active process in which cytoskeletal proteins are involved in the induction of apoptosis-mediated cell fragmentation. In addition, cells constantly release small membrane vesicles of endocytic origin termed exosomes, which are actively secreted upon late endosome fusion with the plasma membrane [65]. Exosomes are mediators of intercellular communication that can deliver information from cell to cell [66]. Sixty-seven genes were up-regulated 20 hours after irradiation, and these may be associated with endosomes or lysosomes with known function in exosomal signaling (e.g., CD68), indicating that IR affects the biological process of extracellular vesicle formation. Exosomes released from irradiated cells have been shown to exert bystander effects, as they induce genomic instability in non-irradiated cells [67]. Therefore, our data suggest that IR is not only a trigger for the induction of apoptotic body formation, but also for the production and release of small extracellular vesicles.

## Conclusion

In this study, we comprehensively analyzed IR-induced changes in mRNA, miRNA, and protein levels and identified p53 as the major early response transcription factor regulated by IR. The miRNA target analysis identified the involvement of miRNAs in the regulation of IR-induced biological processes, and we discovered previously unknown miRNAs involved in the response to IR. We described a complex interaction network of miRNAs and target mRNAs that will encourage future studies to examine the contribution of the specific newly identified miRNAs on the regulation of biological processes in response to IR in more detail.

### Availability of supporting data

The data sets supporting the results of this article are included within the additional files and are available in the LabArchives (https://mynotebook.labarchives.com/) repository using DOI http://dx.doi.org/10.6070/H40V89S4. Gene expression data are available on the Gene Expression Omnibus (GEO) website (http://www.ncbi.nlm.nih.gov/geo/) under accession number GEO: GSE55955.

## Electronic supplementary material

Additional file 1: **PCR primer sequences.** (XLSX 10 KB)

Additional file 2: **mRNAs differentially expressed in irradiated vs. non-irradiated PBMCs.** Differentially expressed mRNAs from irradiated vs. non-irradiated human PBMCs 2 (Datasheet A), 4 (Datasheet B), and 20 hours (Datasheet C) after irradiation are listed. (XLSX 988 KB)

Additional file 3: **Venn diagram of differentially expressed genes.** Changes in the mRNA of irradiated human PBMCs incubated for 2, 4, and 20 hours are shown. (A) The overlap of up-regulated genes with significant expression changes after irradiation. Five genes were up-regulated at all time points. (B) The overlap of down-regulated genes with significant expression changes after irradiation. 25 genes were down-regulated at all time points. (PDF 259 KB)

Additional file 4: **Differentially expressed core genes.** Genes that were significantly up- or down-regulated (BH Corrected p-value <0.05, FC ≥1.5 in 2 of 4 samples) 2, 4, and 20 hours after irradiation are sorted by highest average FC. The values indicate the geometric mean of the FC value of irradiated samples compared to time-matched controls calculated for four different PBMC preparations. The Benjamini Hochberg FDR-corrected p-value is shown. FC = Fold Change. (XLSX 11 KB)

Additional file 5: **Principle component analysis (PCA) of selected genes.** Thirty-one transcripts differentially expressed at all time points are shown with respect to the first three components and are colored with regard to radiation and time point. PCA allows visual identification of data patterns and highlights similarities and differences between samples. PCA was performed using GeneSpring and was based on conditions. All conditions can be clearly separated from each other. Irradiated cells located in three clusters significantly separated from non-irradiated cells. In contrast, 2 and 4 hours after irradiation, non-irradiated cells clustered next to naïve cells, and 20 hours after irradiated cells clustered above these three clusters. (PDF 156 KB)

Additional file 6: **KEGG pathway and GO analysis of up-regulated genes 2 hours after irradiation.** The KEGG pathway (Datasheet A) and enrichment of GO terms (Datasheet B) in up-regulated genes in irradiated PBMCs 2 hours after irradiation were analyzed using the WEBGESTALT analysis tool. All values are sorted by increasing p-values. (XLSX 14 KB)

Additional file 7: **KEGG pathway and GO analysis of up-regulated genes 4 hours after irradiation.** The KEGG pathway (Datasheet A) and enrichment of GO terms (Datasheet B) in up-regulated genes in irradiated PBMCs 4 hours after irradiation were analyzed using the WEBGESTALT analysis tool. All values are sorted by increasing p-values. (XLSX 13 KB)

Additional file 8: **KEGG pathway and GO analysis of up-regulated genes 20 hours after irradiation.** The KEGG pathway (Datasheet A) and enrichment of GO terms (Datasheet B) in up-regulated genes in irradiated PBMCs 20 hours after irradiation were analyzed using the WEBGESTALT analysis tool. All values are sorted by increasing p-values. (XLSX 17 KB)

Additional file 9: **p53 protein interaction network in irradiated cells.** The predicted functional interaction of eight significantly up-regulated transcripts in irradiated PBMCs at two of three time points clustered in the canonical pathway “p53 signaling” were visualized using String v9.1 software. A direct interaction of 16 transcripts is evident. p53 and its downstream target MDM2 are in the center position, interacting with nine partner proteins. Linking line colors are based on their origin: yellow – text mining linkage; blue – database; pink – experiments; black – co-expression. (PDF 4 MB)

Additional file 10: **KEGG pathway and GO analysis of down-regulated genes 2 hours after irradiation.** The KEGG pathway (Datasheet A) and enrichment of GO terms (Datasheet B) in down-regulated genes in irradiated PBMCs 2 hours after irradiation were analyzed using the WEBGESTALT analysis tool. All values are sorted by increasing p-values. (XLSX 12 KB)

Additional file 11: **KEGG pathway and GO analysis of down-regulated genes 4 hours after irradiation.** The KEGG pathway (Datasheet A) and enrichment of GO terms (Datasheet B) in down-regulated genes in irradiated PBMCs 4 hours after irradiation were analyzed using the WEBGESTALT analysis tool. All values are sorted by increasing p-values. (XLSX 12 KB)

Additional file 12: **KEGG pathway and GO analysis of down-regulated genes in irradiated PBMCs 20 hours after irradiation.** The KEGG pathway (Datasheet A) and enrichment of GO terms (Datasheet B) in down-regulated genes in irradiated PBMCs 20 hours after irradiation were analyzed using the WEBGESTALT analysis tool. All values are sorted by increasing p-values. (XLSX 12 KB)

Additional file 13: **Up-regulated KEGG pathway and GO analysis and down-regulated KEGG pathway (Datasheet C) and GO analysis (Datasheet D) in irradiated PBMCs at two of three time points.** The KEGG pathway (Datasheet A and C) and enrichment of GO terms (Datasheet B and D) in up-regulated (Datasheet A and B) and down-regulated genes (Datasheet C and D) in irradiated PBMCs at two of three time points after irradiation were analyzed using the WEBGESTALT analysis tool. All values are sorted by increasing p-values. (XLSX 21 KB)

Additional file 14: **miRNAs differentially expressed in irradiated vs. non-irradiated PBMCs.** Differentially expressed miRNA in human PBMCs 4 (Datasheet A) and 20 hours (Datasheet B) after irradiation are shown. (XLSX 26 KB)

Additional file 15: **Seven up-regulated miRNAs 20 hours after IR and their negatively correlated target genes.** Using Sylamer analysis, miR-887, miR-1306, miR-1180, miR-1268, miR-371-5p, miR-630, and miR-595 exhibited enrichment of seed regions in the set of mRNAs down-regulated 20 hours after IR. Each miRNA, its target genes, and the number of seed regions within each gene are shown. (XLSX 23 KB)

Additional file 16: **Six down-regulated miRNAs 20 hours after IR and their negatively correlated target genes.** Using Sylamer analysis, miR-1237, miR-30a, miR-598, miR-601, miR-205*, and miR-328 exhibited enrichment of seed regions in the set of mRNAs up-regulated 20 hours after IR. Each miRNA, its target genes, and the number of seed regions within each gene are shown. (XLSX 33 KB)

Additional file 17: **KEGG pathways and GO analysis of up-regulated mRNAs controlled by miRNAs 20 hours after irradiation.** 140 annotated genes up-regulated 20 hours after irradiation were classified into different canonical pathways according to KEGG pathway analysis (Datasheet A) or GO analysis (Datasheet B). (XLSX 15 KB)

Additional file 18: **Up-regulated genes targeted by down-regulated miRNAs involved in apoptosis.** The given mRNAs were up-regulated 20 hours after irradiation but their targeting miRNAs were down-regulated. The genes were clustered according to their biological function: apoptosis. The statistic column lists the number of reference genes in the category (C), number of genes in the gene set and the category (O), expected number in the category (E), ratio of enrichment (R), p-value from the hypergeometric test (rawP), and the p-value adjusted by the multiple test adjustment (adjP). For mRNA, the gene symbol, Entrez gene ID, and value expressed as log2 (sample/control) are given. The corresponding miRNA, intensity value expressed as log2 (sample/control), and correlation coefficient are stated. (XLSX 11 KB)

Additional file 19: **Up-regulated genes targeted by down-regulated miRNAs involved in endocytosis.** The given mRNAs were up-regulated 20 hours after irradiation but their targeting miRNAs were down-regulated. The genes were clustered according to their biological function: endocytosis. The statistic column lists the number of reference genes in the category (C), number of genes in the gene set and the category (O), expected number in the category (E), ratio of enrichment (R), p-value from the hypergeometric test (rawP), and the p-value adjusted by the multiple test adjustment (adjP). For mRNA, the gene symbol, Entrez gene ID, and the value expressed as log2 (sample/control) are given. The corresponding miRNA, intensity value expressed as log2 (sample/control), and correlation coefficient are stated. (XLSX 11 KB)

Additional file 20: **KEGG pathways and GO analysis of down-regulated mRNAs controlled by miRNAs 20 hours after irradiation.** 208 annotated genes down-regulated 20 hours after irradiation were classified into different canonical pathways according to KEGG pathway analysis (Datasheet A) or GO analysis (Datasheet B). (XLSX 13 KB)

Additional file 21: **Down-regulated genes targeted by up-regulated miRNAs involved in the cell cycle.** The given mRNAs were down-regulated 20 hours after irradiation but their targeting miRNAs up-regulated. The genes were clustered according to their biological function: cell cycle. The statistic column lists the number of reference genes in the category (C), number of genes in the gene set and the category (O), expected number in the category (E), ratio of enrichment (R), p-value from the hypergeometric test (rawP), and the p-value adjusted by the multiple test adjustment (adjP). For mRNA, the gene symbol, Entrez gene ID, and value expressed as log2 (sample/control) are given. The corresponding miRNA, intensity value expressed as log2 (sample/control), and correlation coefficient are stated. (XLSX 11 KB)

Additional file 22: **Bioinformatics analysis of putative transcription factor binding sites (oPOSSUM3 single site binding analysis method) in up-regulated genes 2, 4, and 20 hours after radiation.** Significant results are shaded in red. (XLSX 30 KB)

## Abbreviations

PCA: Principle component analysis
GO: Gene ontology
GEO: Gene expression omnibus
IR: Ionizing radiation
mRNA: Messenger RNA
miRNA: Micro RNA
KEGG: Kyoto Encyclopedia of Genes and Genomes
PBMC: Peripheral blood mononuclear cells
qPCR: Quantitative real-time PCR
TF: Transcription factor
TFBS: Transcription factor binding site.
