# Supplementary figures and images for: High dose ionizing radiation regulates micro RNA and gene expression changes in human peripheral blood mononuclear cells
Source: BMC Genomics. 2014 Sep 25;15(1):814. doi: 10.1186/1471-2164-15-814 (PMC4182888; doi:10.1186/1471-2164-15-814)

Additional file 3

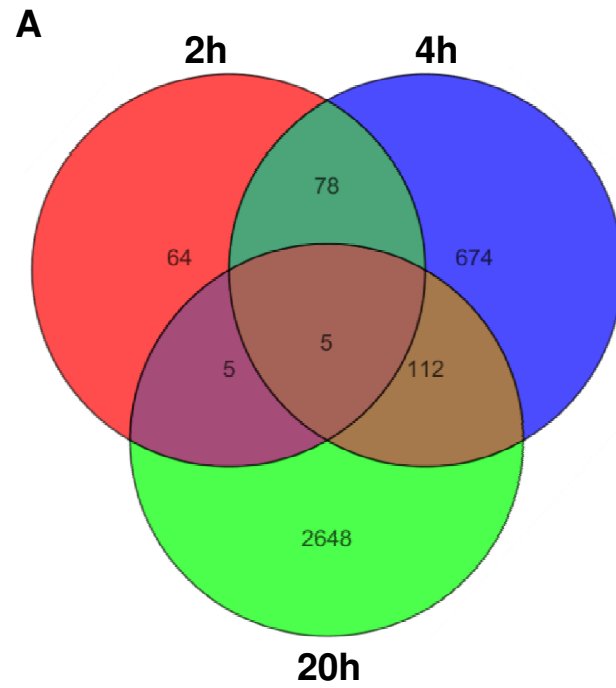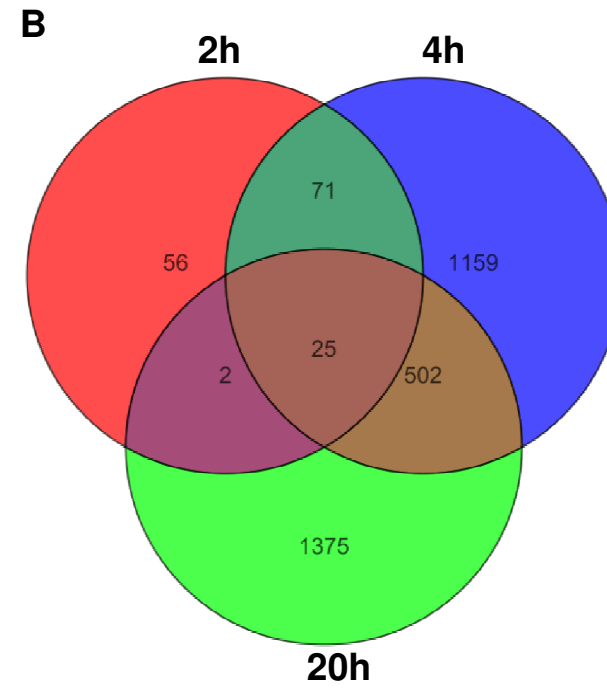

Supplement: Supplementary file 3 — Additional file 3: Venn diagram of differentially expressed genes. Changes in the mRNA of irradiated human PBMCs incubated for 2, 4, and 20 hours are shown. (A) The overlap of up-regulated genes with significant expression changes after irradiation. Five genes were up-regulated at all time points. (B) The overlap of down-regulated genes with significant expression changes after irradiation. 25 genes were down-regulated at all time points. (PDF 259 KB) [file 12864_2014_6490_MOESM3_ESM.pdf]

## Additional file 5

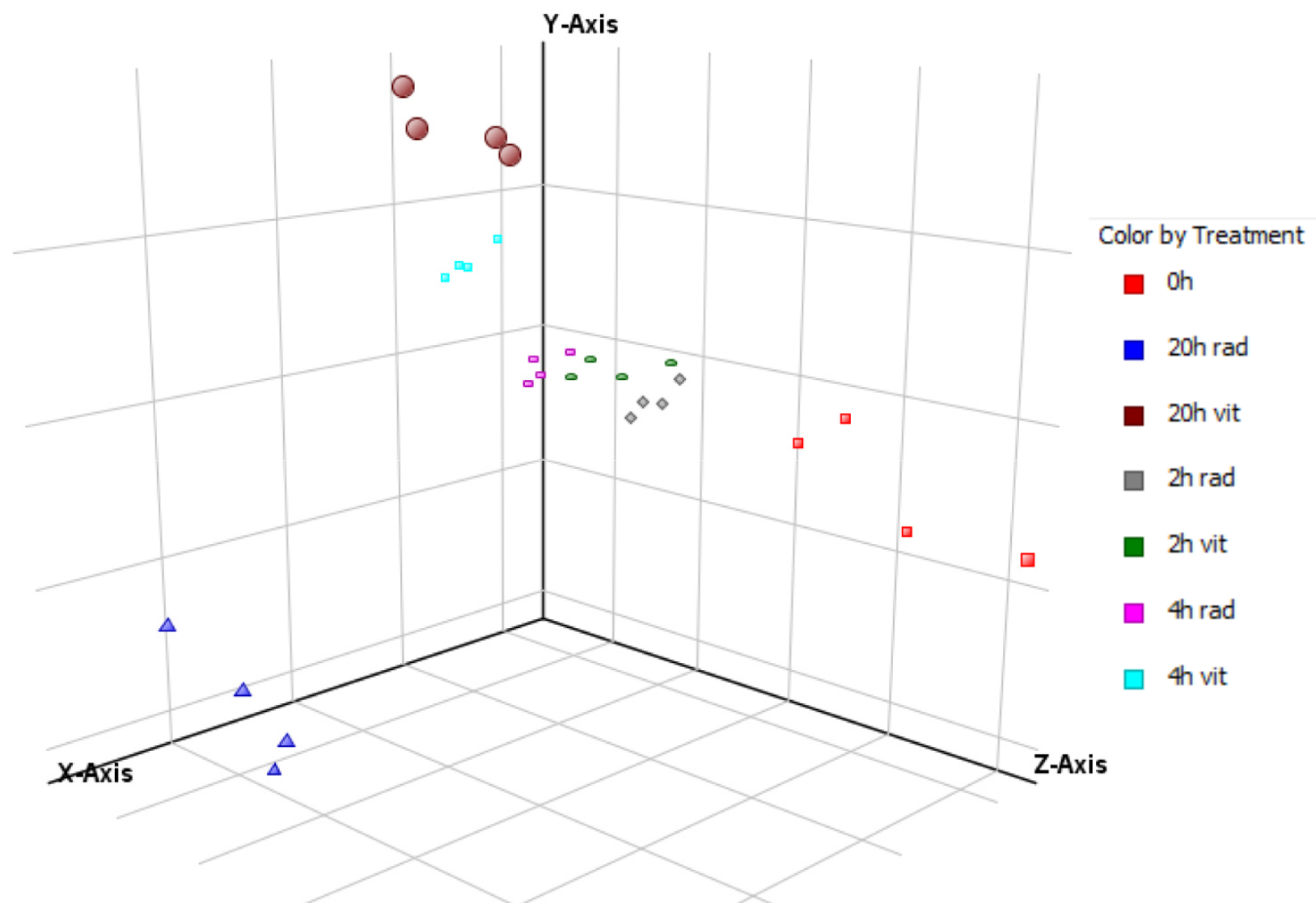

Supplement: Supplementary file 5 — Additional file 5: Principle component analysis (PCA) of selected genes. Thirty-one transcripts differentially expressed at all time points are shown with respect to the first three components and are colored with regard to radiation and time point. PCA allows visual identification of data patterns and highlights similarities and differences between samples. PCA was performed using GeneSpring and was based on conditions. All conditions can be clearly separated from each other. Irradiated cells located in three clusters significantly separated from non-irradiated cells. In contrast, 2 and 4 hours after irradiation, non-irradiated cells clustered next to naïve cells, and 20 hours after irradiated cells clustered above these three clusters. (PDF 156 KB) [file 12864_2014_6490_MOESM5_ESM.pdf]

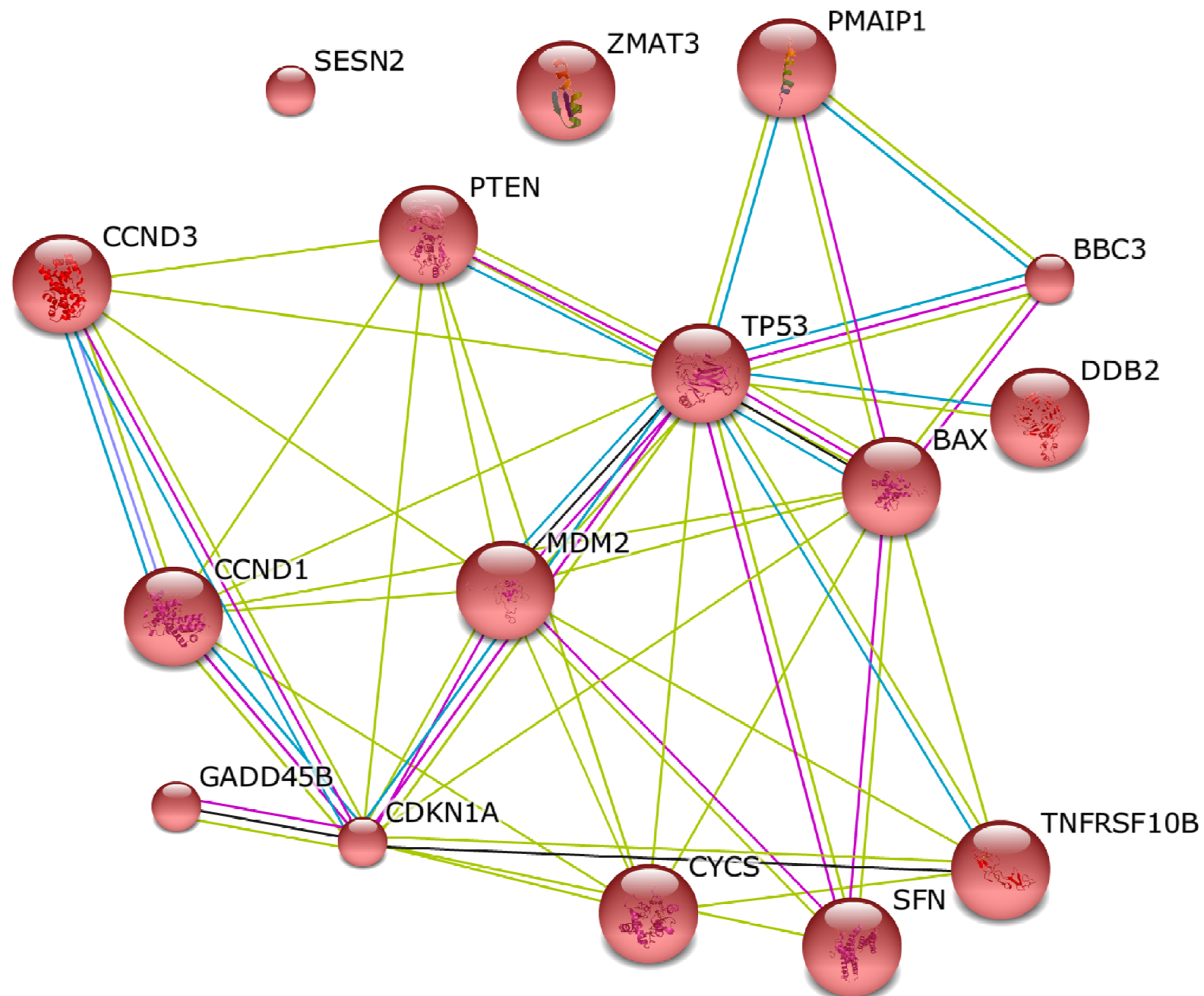

Supplement: Supplementary file 9 — Additional file 9: p53 protein interaction network in irradiated cells. The predicted functional interaction of eight significantly up-regulated transcripts in irradiated PBMCs at two of three time points clustered in the canonical pathway “p53 signaling” were visualized using String v9.1 software. A direct interaction of 16 transcripts is evident. p53 and its downstream target MDM2 are in the center position, interacting with nine partner proteins. Linking line colors are based on their origin: yellow – text mining linkage; blue – database; pink – experiments; black – co-expression. (PDF 4 MB) [file 12864_2014_6490_MOESM9_ESM.pdf]
